# Treatment of Hepatitis C Infections with Interferon: A Historical Perspective

**DOI:** 10.1155/2010/323926

**Published:** 2010-09-06

**Authors:** Robert M. Friedman, Sara Contente

**Affiliations:** Department of Pathology, Uniformed Services University of the Health Sciences, Bethesda, MD 20814, USA

## Abstract

Interferons were first described in 1957, but it was not until 34 years after their discovery that sufficient quantities of it were available for treatment of hepatitis C virus (HCV) infections, Clinicians now have an excellent understanding of the basis for the effectiveness of interferon alpha (IFN-*α*) in the therapy of this disease. Treatment with IFN-*α* is more efficient when it complemented by the antiviral ribavirin and the IFN-*α* is conjugated with polyethylene glycol to form peginterferon. In the near future treatment of HCV with IFN-*α* may involve new anti-HCV agents that are currently under development.

The antiviral activity of interferon (IFN), first described in 1957, was in a chick cell and inactivated influenza virus system [1]. The inactivated virus induced a protein that had a broad spectrum of antiviral activity, which immediately attracted wide interest, so that there was expectation that interferons (IFNs) rapidly would develop clinically as agents to treat a range of viral infections. In addition to their antiviral activity, IFNs were later discovered to be important regulators of both cellular growth and the immune response. A number of problems arose, however, that delayed their clinical use for the treatment of virus infections for many years. The first of these was that the IFNs, with some exceptions, are species-specific in their biological activity [2], so that only human or primate interferons were found to be active in humans. This meant that the single source of interferons for human use in the 1960s and 70s was primate cells, and the supply of such cells was quite limited. Another problem was that IFNs could only be assayed by means of their ability to inhibit virus replication in a tissue culture system [1]. In addition, IFNs were found to possess then unprecedented biological activity, and it became evident that existing stocks of IFNs with very significant antiviral activity actually were quite impure and so contained very little IFN. Because of the lack of even moderately clean IFN, it was impossible to accept any biological activity of an IFN preparation, other than antiviral activity, as being due to its IFN content, although subsequently IFNs were shown to have many biological functions. Despite such problems, and because of the promise IFNs held as a possible treatment for viral diseases, there were early clinical trials of the antiviral activity of what IFN preparations were then available. These studies tested the ability of an IFN produced by simian cells to inhibit the development of vaccinia virus lesions in human skin or respiratory infections following exposure of volunteers to common cold viruses [3, 4]. The results were unimpressive, almost certainly because of the small quantities of impure IFN used, so that for many years studies on IFNs were limited to experiments in tissue culture and to attempts to produce and purify sufficient quantities of IFN from human cells to carry out significant clinical studies. To further complicate matters, it was discovered that there were actually several forms of human IFN, IFNs-*α*, -*β*, and -*γ*. There are seven subtypes of human IFN-*α*, but only single genes coding for IFNs-*β* and -*γ*. Subsequently, additional forms were discovered, but only IFNs -*α*, -*β*, and -*γ* are presently used clinically.

 Interest in IFNs was reignited in the mid-1970s when sufficient quantities of fairly clean human IFN-*α*, obtained by Cantell's group in Finland from the white blood cell buffy coats of donated blood, became available [5] for clinical experiments. Many of these had promising, if not highly significant, results in studies on the prevention of common colds [6] and the treatment of several herpes virus infections, such as herpes keratoconjunctivitis and the varicella-zoster infections, shingles and chickenpox [7, 8]. The discovery that in tissue culture experiments mouse IFN-*β* inhibited chronic infections with mouse leukemia viruses [9] prompted additional studies employing interferon as therapy for human chronic hepatitis B virus (HBV) infections. These had very promising results [10]. 

 A 1974 report that Cantell's IFN-*α* was an effective treatment for cancer, although later shown to be flawed, nevertheless had profound effects on interferon research, both positive and negative [11]. That IFNs might be potential anticancer drugs led to widespread, unwarranted, and later disappointed expectations of their being a general cure for cancer; on the positive side, however, interest in finding better sources for a potential wonder drug led directly to the cloning of genes for human IFN-*α* [12], and later for IFNs-*β* and -*γ* [13, 14]. This in turn led to the production of quantities of pure IFNs sufficient to carry out a large number of clinical trials with significant results. Such studies have partially clarified what the role of IFNs might be in the treatment of several diseases. Recombinant IFN-*α*s presently are widely employed with some success in the treatment of chronic hepatitis B virus (HBV) and hepatitis C virus (HCV) infections and with limited effectiveness, in some forms of neoplasia such as melanomas [15]. IFN-*β* treatment is regularly used to limit exacerbations of multiple sclerosis [16]. IFN-*γ* has been approved for clinical use only in a rare congenital disorder, chronic granulomatous disease, for which it is effective in preventing recurrent bacterial infections. Current clinical trials are underway employing in the treatment of chronic HBV and HCV infections IFN-*λ*, which is biologically similar to IFNs-*α* and -*β*, but employs a different membrane receptor [17, 18]. Phase 1 trials for IFN-*λ* were successfully completed in October, 2009, and Phase 2 trials have been initiated. 

By far the best understood clinical application of IFNs biologically is against chronic HCV infections, for which IFN-*α* has been an approved treatment since 1991, although IFN treatment for HCV was first employed in 1986 with some promise, well before the viral cause of the infection had been identified [19, 20]. HCV is a widespread infection spread by contaminated blood products or by drug injection. Although modern blood bank technology has almost eliminated the former, the latter remains a major problem. There are worldwide millions of HCV-infected patients. The progress of HCV infections is insidious, often not being clinically manifest for two or three decades after initial infection with the virus. Chronic HCV infection may cause serious hepatic malfunction eventually resulting in cirrhosis of the liver and in life-threatening esophageal varices. In addition, a significant number of patients with chronic HCV infections eventually develop hepatocellular cancers (hepatomas) and have an increased risk for developing renal cell carcinomas [21]. Chronic infections with HCV are a significant cause of death in patients with AIDS [22]. 

HCV is a small Flavivirus, the sole member of the hepacivirus ribovirus species, with seven genotypes, of which genotype 1, unfortunately the most common infection in North America, is relatively insensitive to IFN-*α*. It appears possible to predict the response of a patient to infection with a genotype 1 HCV isolate by use of structural analysis of the infecting virus [23]. The core protein of genotype 1 HCV induces cellular proliferation and transformation and so is associated with advanced hepatic cirrhosis and hepatocellular transformation [24]. The resistance of HCV to IFN resides in a nonstructural viral protein NS3/4A, a serine protease that inactivates the signal leading to interferon production, thus apparently facilitating the development of chronic infections [25]. IFNs-*α* and -*β* production is induced when a cellular protein receptor, RIG-1 (retinoic acid inducible gene), is activated by single-strand virus RNA. Activated RIG-1 in turn interacts with the adaptor mitochondrial antiviral signaling (MAVS) protein that phosphorylates IFN response factor 3 (IRF3), leading eventually to production of IFN [26]. The viral NS3/4 protease inhibits interferon production by hydrolyzing the attachment of MAVS to its site on mitochondria. HCV growth is, however, sensitive to the antiviral action of IFN although the mechanism for this inhibition is presently uncertain. It may involve two of the proteins induced by IFN treatment, a ribonuclease that destroys HCV RNA or a protein kinase that inactivates a factor required for virus protein synthesis [27]. The expression of the gene for IL-28B, which codes for IFN-*λ*, is a predictor of the ability of patients to clear HCV or to respond to therapy for HCV infections [28]. Patients with severe cases of HCV appear to respond to IFN therapy better than do patients with more moderate infections [29]. 

In order to augment the effectiveness of IFN-*α* employed in the treatment of HCV, two alterations in the protocol for its treatment were initiated. Ribavirin, an oral purine analogue that inhibits the growth of some RNA viruses, such as flaviviruses, either by inhibiting the HCV polymerase or by inducing lethal virus mutations among other possible mechanisms, was added to the regimen [30]; and IFN-*α* was conjugated to polyethylene glycol to yield peginterferon. This conjugation decreases the renal clearance of the IFN and so significantly increases its half-life from about 5 h to almost 90 h, which in turn allows a reduction in the required frequency of treatments [31, 32]. Of the two forms of peginterferon available, peginterferon alfa-2a appears to be somewhat more effective than does peginterferon alfa-2b [33, 34]. With the combined ribavirin/peginterferon treatment, more than 75% of nongenotype 1 HCV patients maintain a sustained anti-HCV response, and up to 50% of the patients infected with the genotype 1 HCV responded to this combined treatment; in those patients responding to peginterferon/ribavirin therapy, virus-induced liver damage failed to progress, with some degree of healing taking place [31]. IFN-based treatment was associated with improved survival and reduced the risk of hepatocellular cancer. Long-term followup indicated that once a particular HCV-infected patient attains a sustained response to peginterferon/ribavirin therapy, defined as undetectable levels of HCV RNA in the serum for six months, the risk for virologic relapse is very low [35]. In one clinical study, low doses of peginterferon and ribavirin were as effective as higher dose levels [24, 36]. 

Current treatments for chronic HCV infections have several limitations, as they result in rates of sustained virus responses that were lower in black and Latino patients than in non-Latino whites [37, 38]. Long-term, IFN-based treatment did not halt the progression of chronic HCV infections in patients not responding to initial treatment [36]. A variable percentage of patients treated with IFN develop anti-IFN antibodies, but surprisingly, there appears to be little correlation between the presence of such antibodies and the response to IFN [39]. IFN-*α* is also useful in the treatment of cryoglobulinemia and focal glomerulopathy, complications of chronic HCV infections [40].

 Unfortunately, the prolonged peginterferon therapy necessary to control chronic HCV or HBV infections was often associated with serious side effects such as fatigue, fever, and myalgias, symptoms of many acute virus infections, possibly because such effects are due to the induction of IFNs by the infecting agents. Usually these symptoms respond to treatment with nonsteroidal anti-inflammatory agents [27]. In some patients, treatment with IFNs has also resulted in psychiatric problems such as depression, anxiety, and excessive irritability that may require treatment with psychoactive pharmaceuticals. More severe toxicities, such as cytopenias and autoimmune disorders, also have rarely been reported in patients treated with IFNs [41].

In patients who did not respond to standard peginterferon/ribavirin therapy, substitution of the consensus interferon, alfacon-1, plus ribavirin proved effective in some cases [42]. Currently, new forms of therapy to augment treatment with ribavirin/peginterferon are under development, including inhibitors of the HCV protease, helicase, or polymerase and IFN-*α* conjugated to albumin [43]. Telaprevir, an inhibitor of the HCV nonstructural protease NS3/4, has proven to be effective when employed with peginterferon/ribavirin to treat patients with chronic HCV infections that are unresponsive to conventional peginterferon/ribavirin therapy [44]. Combinations of additional new agents with the currently employed therapies may provide effective treatment for a much larger percentage of HCV patients than are currently responding to anti-HCV treatment [31].
